# Domestic violence in Tunisia: which forms of physical violence?

**DOI:** 10.1192/j.eurpsy.2024.1681

**Published:** 2024-08-27

**Authors:** R. Jbir, L. Aribi, I. Chaari, F. Guermazi, A. Samet, N. Bouattour, N. Messedi, J. Aloulou

**Affiliations:** psychiatry B, Hedi chaker hospital university, Sfax, Tunisia

## Abstract

**Introduction:**

Domestic violence is a universal phenomenon that destroys the fabric of society and threatens the lives, health and prosperity of all.

It can take different forms, including physical abuse. This is one of the most serious form of violence, as it can range from a simple shove to homicide.

**Objectives:**

To determine the prevalence and describe the various forms of physical violence perpetrated by husbands against their wives.

**Methods:**

We contacted women who consulted at the psychiatric emergency of ‘Hedi Chaker hospital’,Sfax examined in the context of medical expertise on the period between May 2021 until January 2022.

A questionnaire regarding the violence was asked to responders. It included a section for collecting socio-demographic and clinical data on the woman, and a section for assessing the various forms of domestic violence.

**Results:**

122 women were surveyed. The average age of victims was 35.66 years with extremes of 18 and 64 years. 78.7% (n=96) of ladies were of urban origin. The majority of them (44,3%) had secondary level education.

The half of the population (51.6%) had an average socio-economic level and 43.4% (n=53) lived in rented houses.

All the women of our population were married: it was the first marriage in (89.3%) and the majority (86.1%) had children.

Almost all women (95.1%; n=116) were victims of physical violence.

Different types of physical violence were reported with decreasing prevalence: slap (65,6%), punch (58,2%), strangle (46,7%), kicking (38,1%), stabbing threat (28,7%), kidnapping (4,9%), and gun threat (3,3%).

Should be noted that some women experience different forms of violence simultaneously.

**Conclusions:**

Our study showed a high prevalence of physical violence with different shapes.

These figures must be taken into account by the authorities given the gravity of physical and psychological consequences of this form of violence.

**Disclosure of Interest:**

None Declared

